# Circulating Endothelial-Derived Activated Microparticle: A Useful Biomarker for Predicting One-Year Mortality in Patients with Advanced Non-Small Cell Lung Cancer

**DOI:** 10.1155/2014/173401

**Published:** 2014-06-29

**Authors:** Chin-Chou Wang, Chia-Cheng Tseng, Chang-Chun Hsiao, Huang-Chih Chang, Li-Teh Chang, Wen-Feng Fang, Steve Leu, Yi-Hsi Wang, Tzu-Hsien Tsai, Cheng-Ta Yang, Chih-Hung Chen, Hon-Kan Yip, Chi-Kung Ho, Meng-Chih Lin

**Affiliations:** ^1^Division of Pulmonary and Critical Care Medicine, Department of Internal Medicine, Kaohsiung Chang Gung Memorial Hospital, Chang Gung University College of Medicine, Kaohsiung 83301, Taiwan; ^2^Department of Public Health, Kaohsiung Medical University, Kaohsiung 807, Taiwan; ^3^Department of Respiratory Care, Chang Gung University of Science and Technology, Chiayi Campus, Chiayi City 613, Taiwan; ^4^Graduate Institute of Clinical Medical Sciences, Chang Gung University College of Medicine, Kaohsiung 83301, Taiwan; ^5^Roswell Park Cancer Institute, Buffalo, NY 14263, USA; ^6^Basic Science, Nursing Department, Meiho University, Pingtung 900, Taiwan; ^7^Center for Translational Research in Biomedical Sciences, Kaohsiung Chang Gung Memorial Hospital and Chang Gung University College of Medicine, Kaohsiung 83301, Taiwan; ^8^Division of Cardiology, Department of Internal Medicine, Kaohsiung Chang Gung Memorial Hospital and Chang Gung University College of Medicine, Kaohsiung 83301, Taiwan; ^9^Department of Pulmonary and Critical Care Medicine, Chang Gung Memorial Hospital, Chang Gung University College of Medicine, Taoyuan 333, Taiwan; ^10^Divisions of General Medicine, Kaohsiung Chang Gung Memorial Hospital and Chang Gung University College of Medicine, Kaohsiung 83301, Taiwan

## Abstract

*Background*. This study tested the hypothesis that circulating microparticles (MPs) are useful biomarkers for predicting one-year mortality in patients with end-stage non-small cell lung cancer (ES-NSCLC). *Methods and Results*. One hundred seven patients were prospectively enrolled into the study between April 2011 and February 2012, and each patient received regular follow-up after enrollment. Levels of four MPs in circulation, (1) platelet-derived activated MPs (PDAc-MPs), (2) platelet-derived apoptotic MPs (PDAp-MPs), (3) endothelial-derived activated MPs (EDAc-MPs), and (4) endothelial-derived apoptotic MPs (EDAp-MPs), were measured just after the patient was enrolled into the study using flow cytometry. Patients who survived for more than one year were categorized into group 1 (*n* = 56) (one-year survivors) and patients who survived less than one year were categorized into group 2 (*n* = 51) (one-year nonsurvivors). Male gender, incidence of liver metastasis, progression of disease after first-line treatment, poor performance status, and the Charlson comorbidity index were significantly higher in group 2 than in group 1 (all *P* < 0.05). Additionally, as measured by flow cytometry, only the circulating level of EDAc-MPs was found to be significantly higher in group 2 than in group 1 (*P* = 0.006). Multivariate analysis demonstrated that circulating level of EDAc-MPs along with brain metastasis and male gender significantly and independently predictive of one-year mortality (all *P* < 0.035). *Conclusion*. Circulating EDAc-MPs may be a useful biomarker predictive of one-year morality in ES-NSCLC patients.

## 1. Introduction

Clinical observational studies have demonstrated that lung cancer remains the leading cause of cancer-related mortality worldwide, with nearly 1.4 million deaths each year [1–4]. These studies further demonstrated that, in many parts of the world, the number of cases and deaths related to lung cancer is rising [1]. Non-small cell lung cancer (NSCLC), which includes adenocarcinoma, squamous cell carcinoma, large cell carcinoma, and bronchioloalveolar carcinoma, accounts for more than 80% of all cases of lung cancer [2]. Despite concerted efforts to improve survival rate with combinations of traditional surgical intervention and adjunctive radiation and chemotherapy, as well as molecularly targeted drugs such as tyrosine kinase inhibitors of EGFR gene (gefitinib and erlotinib) [5] and tyrosine kinase inhibitors of anaplastic lymphoma kinase (ALK) gene (crizotinib) [6], the overall survival rate is still poor [7–10].

Although advanced molecular biology techniques have greatly accelerated the understanding of cancer biology, still approximately 50% of the patients with lung cancer have advanced stage disease at the time of diagnosis [2]. Thus, not only a better understanding of the pathogenesis of LC, but also the development of molecular and cellular biomarkers [2] for early detection is very important for improving LC therapeutic outcome [11–13]. The development of a serum biomarker that can be applied simply and easily in clinical practice as a complementary tool for the prediction of prognostic outcome would be a welcome addition in the LC setting.

Microparticles (MPs) are “small plasma membrane fragments” of cells released into circulation in response to many situational changes such as physiological conditions, microenvironmental stimulation, stress, physical activation (thrombin, endotoxin, or shear stress), activated cells or those undergoing apoptosis (growth factor deprivation or apoptotic inducers), ischemic stimulations, or neoplastic transformation [14]. MPs are small membrane-enclosed vesicles that are derived from the cellular plasma membrane and shed into circulation by activated and/or apoptotic cells [14–19]. MPs are extremely small-ranging in size from 0.1 *μ*m to 1.0 *μ*m. Interestingly, MPs have been previously reported to participate in inflammation and thrombosis formation [14–18]. In addition, MPs have been identified to have differential roles in angiogenesis depending on their origin [14, 17, 20–23]. Of note, MPs from platelets (PMPs) have been demonstrated to exhibit proangiogenic activity; that is, they promote capillary-like structures and proangiogenic factor production [14, 17, 20, 22]. Conversely, endothelial-derived MPs can be pro- or antiangiogenic depending on the stimuli used for their production [14].

Accumulating published data demonstrates that numbers of circulating MPs are increased in a wide range of diseases [14, 24–30], including LC [31]. We also recently demonstrated that circulating levels of MPs are significantly increased in LC patients, and circulating levels of endothelial-derived apoptotic MPs (EDAp-MPs) are significantly associated with different LC cell types [32]. However, whether circulating level of MPs could be a useful biomarker for prediction of one-year mortality in patients with end-stage NSCLC (ES-NSCLC) remains uncertain. To extend our recent study [32], the aim of this study was to test (1) whether circulating levels of MPs are significantly lower in ES-NSCLC patients who survived for more than one year (defined as one-year survivors) than those patients who survived for less than one year (defined as one-year nonsurvivors) and (2) whether circulating level of MPs is significantly and independently predictive of one-year mortality.

## 2. Materials and Methods

### 2.1. Patient Enrollment, Data Collection, and Therapeutic Strategy

The procedure and protocol of patient enrollment, data collection, classification of ES-NSCLC, and therapeutic strategy were based on the protocol outlined in our recent report [32]. In brief, image studies and pathological findings of all patients who received evaluation or treatment with LC at Kaohsiung Chang Gung Memorial Hospital were assessed, and patients' eligibility for intervention, including surgery, adjunctive/palliative chemotherapy, irradiation therapy, and/or target therapy, was evaluated based on the AJCC Cancer Staging Manual (7th edition) criteria [33]. Lung cancer was categorized into stages I, II, III, and IV according to the radiological findings based on the AJCC Cancer Staging Manual (7th edition) criteria [33]. Additionally, patients who had stage IIIb or stage IV NSCLC upon presentation were categorized into ES-NSCLC. All patients were enrolled either in the outpatient department or upon hospital admission for further evaluation and treatment.

Detailed in-hospital and follow-up data, including age, gender, chest X-ray findings, computed tomography, fibrobronchoscopic findings, bone scans or ultrasound studies, other image findings, and histological and pathological findings, were collected prospectively and entered into a computer database.

One hundred seven consecutive patients of all ages who were diagnosed with ES-NSCLC between April 2011 and February 2012 were prospectively enrolled into the study [stage IIIb, 18.7% (20/107); stage IV, 81.3% (87/107)] (Table 2).

Informed consent was obtained from each study subject. The whole study protocol was approved by the Institutional Review Committee on Human Research at Kaohsiung Chang Gung Memorial Hospital (IRB number: 100-1024B), and clinical investigation was conducted according to the principles outlined in the Declaration of Helsinki.

To circumvent other potential influences on measurement of circulating level of MPs, patients with one or more of the following were excluded [32]: recent surgery or trauma during the preceding 2 months, refusal to participate in the study, other coexistent malignances, severe organ disease other than LC, chronic kidney disease (CKD > stage III), liver cirrhosis, hematologic disorders, congestive heart failure, current use of antiplatelet agents, history of febrile disorders, acute or chronic inflammatory disease other than LC during the study period, or a history of autoimmune diseases with or without immunosuppressive therapy.

### 2.2. Categorization of Circulating Microparticles into Four Types

**Figure 1:**
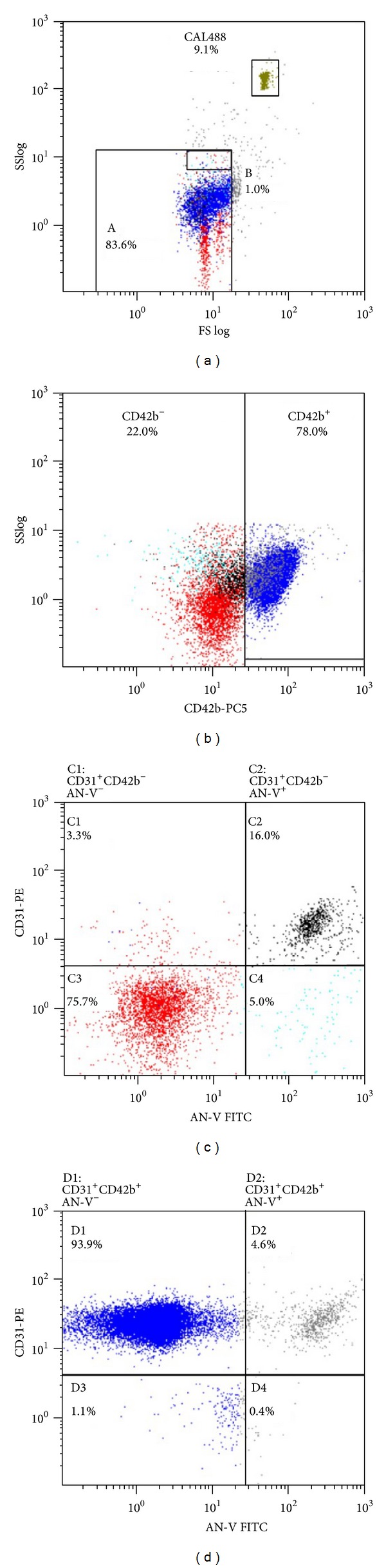
Illustrating one example of flow cytometric result for identification of four different types of microparticles.

Circulating MPs were categorized into (Figure 1) (1) platelet-derived activated MPs (PDAc-MPs) (CD31^+^CD42b^+^AN-V^−^); (2) platelet-derived apoptotic MPs (PDAp-MPs) (CD31^+^CD42b^+^AN-V^+^); (3) endothelial-derived activated MPs (EDAc-MPs) (CD31^+^CD42b^−^AN-V^−^); and (4) endothelial-derived apoptotic MPs (EDAp-MPs) (CD31^+^CD42b^−^AN-V^+^) based on a previous report [34] with some modification and further validated by our recent report [32]. The CD31 biomarker which was chosen as an endothelial cell-derived MPs was basic in previous [34] and our recent [32] reports, as well as the recent report which suggested that CD31^+^ surface marker served as an endothelial cell surface marker [35].

### 2.3. Blood Samples for Biochemical Analysis, Blood Cell Count Study, and Flow Cytometric Analysis for Plasma Levels of Microparticles

Blood samples were obtained once at 9:00 am from study subjects for individual analysis according to the procedure and protocol outlined in our previous study [32]. In brief, white blood cell (WBC) counts, biochemistry, and electrolyte levels were analyzed using standard laboratory methods in our hospital. Peripheral blood was collected in acid citrate dextrose (ACD) vacutainer tubes. To prepare platelet-rich plasma, the peripheral blood (1.5 mL) was centrifuged at 2500 ×g at 4°C for 15 min without acceleration or break. The 250 *μ*L plasma samples were thawed and centrifuged for 10 min at 19,800 ×g at 4°C and then collected for investigation of microparticles (MPs) smaller than 1.0 *μ*m.

Size calibration was conducted with 1.0 *μ*m beads (Invitrogen, Carlsbad, CA). The MP pellet was resuspended with 150 *μ*L of Annexin-V binding buffer (BD Biosciences). All buffers were sterile-filtered with a 0.2 *μ*m filter. The 100 *μ*L MPs were then incubated in a TruCOUNT tube (BD Biosciences) with fluorescent monoclonal antibodies: (1) phycoerythrin- (PE-) labeled anti-CD31 (BD Biosciences); (2) fluorescein isothiocyanate-labeled anti-Annexin-V (BD Biosciences); and (3) phycoerythrin-Cy5- (PE-Cy5-) labeled anti-CD42b (BD Biosciences). The samples were incubated in the dark for 15 min at room temperature. The samples were then analyzed on a FC500 flow cytometer (Beckman Coulter) after 400 *μ*L Annexin-V binding buffer was added. The absolute count of MPs was measured setting the stop condition for TruCount beads at 10,000 events.

### 2.4. Definitions

Assessment of the change in tumor burden which was used to determine whether or not the tumor responded to the adjunctive therapy was conducted based on the protocol outlined in our previous report [32]. Accordingly, chest computed tomography (CT) scans were routinely performed at baseline and at three time points (at 12-week intervals) of adjunctive therapy to determine the status of the disease. The tumor measurement criteria were based on the current Response Evaluation Criteria in Solid Tumors (RECIST) guidelines [36] and included complete response, partial response, stable disease, and progressive disease. In the current study, we categorized the disease statuses as (1) disease controlled, (2) disease progression, and (3) disease without treatment. “Disease controlled” status was defined as disease posttreatment with regression, in a stable condition (i.e., including complete response, partial response, and stable disease). “Disease progression” was defined as disease unresponsive to therapy (complete course of treatment with tumor growing or disease metastasis). “Disease without treatment” was defined as a fresh case that was enrolled prior to treatment.

### 2.5. Statistical Analysis

Data were expressed as means ± SD. Categorical variables were analyzed using the chi-squared test or Fisher's exact test where appropriate and continuous variables were compared using Student's *t*-test or the Mann-Whitney *U* test. All variables were considered as risk factors with a *P* value <0.10 in univariate analysis that were entered into the multivariate model. Multivariate logistic regression analysis was performed to identify independent factors for predicting one-year survival. Receiver operating characteristic (ROC) curves were plotted and the area under the curve was compared with several serum biomarkers measured in this study. The cutoff value of serum biomarkers for predicting one-year survival among lung cancer patients was analyzed according to ROC curves. The proportion of patients over time was estimated by means of Kaplan-Meier analysis comparing subjects identified as independent factors by multivariate method as well as first-line treatment status.

Results were presented as absolute numbers (percentage) or mean ± standard deviation (SD). Odds ratios and 95% confidence intervals (CIs) were reported for logistic regression analysis. A two-tailed *P* value of <0.05 was considered significant. All statistical analysis was performed using the SPSS 14.0 software package (SPSS Inc., Chicago, IL, USA).

## 3. Results

### 3.1. Baseline Characteristics and Clinically Relevant Variables of 107 Study Patients (Table 1)

The age and body mass index did not differ in one-year survivors and one-year nonsurvivors. Additionally, no patient among these two groups had previously received surgical intervention. However, male gender was significantly more prevalent in the population of one-year nonsurvivors than in one-year survivors. The incidences of history of smoking, hypertension, and diabetes mellitus did not differ between these two groups of patients.

Red blood cell count, white blood cell count, and platelet count and the levels of creatinine, aspartate aminotransferase (AST), and alanine aminotransferase (ALT) were similar among these two groups of patients. Additionally, the percentage use of adjunctive therapy, including irradiation therapy, traditional chemotherapy, and target therapy, did not differ between these two groups.

Table 1 also shows the clinical relevant variables in one-year survival and one-year non-survival patients. Subgroup analysis of these ES-NSCLC patients showed no significant difference between the distribution of stage IIIb and stage IV among the one-year survivors and one-year nonsurvivors. Additionally, the incidence of specified metastatic sites, including pleural, intrapulmonary, bone, adrenal gland, and brain was similar between these two groups of patients. However, the incidence of metastasis to the liver was significantly higher in one-year nonsurvivors than in one-year survivors.

To elucidate the disease status after a complete course of the first-line treatment, the parameters, that is, the disease control and disease progression, were carefully assessed. The results showed that the incidence of disease control was significantly higher, whereas the disease progression was significantly lower in one-year survivors than in one-year nonsurvivors.

WHO performance status was used to determine patients' activity capacities [37]. The results showed significantly poorer performance status in one-year nonsurvivors as compared with those of one-year survivors. Additionally, to measure the burden of comorbid diseases in ES-NSCLC patients, MED-ECHO database (Quebec), the so-called Charlson index [38], was adopted. As expected, the Charlson comorbidity index was significantly higher in one-year nonsurvivors than in one-year survivors.

### 3.2. Flow Cytometric Quantification of Circulating MPs Levels among the 107 Study Patients (Table 2)

Table 2 shows the results of flow cytometry for analyzing the circulating levels of MPs. The results show that the circulating levels of PDAc-MPs, PDAp-MPs, and EDAp-MPs did not differ between one-year survivors and one-year nonsurvivors. However, the circulating level of EDAc-MPs was substantially higher in one-year nonsurvivors than in one-year survivors.

### 3.3. Determination of the Predictors of One-Year Mortality among 107 Study Patients (Table 3)

Table 3 shows the univariate and multivariate analysis of predictive factors of one-year mortality. The variables in Tables 1 and 2 were utilized in the statistical analysis in the current study. The results demonstrated that male gender, liver metastasis, and lower performance status were significantly predictive of one-year mortality. Additionally, brain metastasis showed a tendency towards statistical significance for prediction of one-year mortality. Conversely, the lower the Charlson comorbidity index values, the lower the levels of circulating EDAc-MP, and disease control status was significantly associated with one-year survival.

Multiple stepwise-logistic regression analysis showed that, among the four types of MPs, only an increase in circulating level of EDAc-MPs was significantly and independently predictive of one-year mortality. Additionally, male gender and brain metastasis were significant independent predictors of one-year mortality.

### 3.4. Correlation between Circulating Level of EDAc-MPs and One-Year Mortality

**Figure 2:**
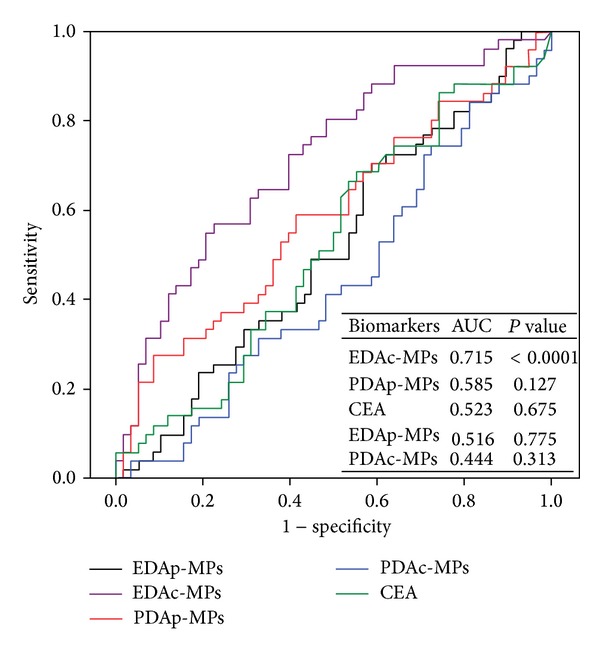
Receiver operating characteristic (ROC) plot of circulating level of microparticles (MPs) predictive of one-year mortality. The ROC curve analysis showed that only circulating level of endothelial-derived activated- (EDAc-) MPs ≥ 1100.5 counts/mL (i.e., cutoff value) was the most powerful predictor of one-year mortality with a sensitivity of 77.6% and a specificity of 56.9%. Area under the curve = 0.715, *P* < 0.0001. MP = microparticles; CEA = carcinoembryonic antigen.

Receiver operating characteristics (ROC) curve analysis (Figure 2) revealed that circulating level of EDAc-MPs ≥1100.5 counts/mL (i.e., cutoff value) was the most powerful predictor of one-year mortality with a sensitivity of 77.6% and a specificity of 56.9%.

**Figure 3:**
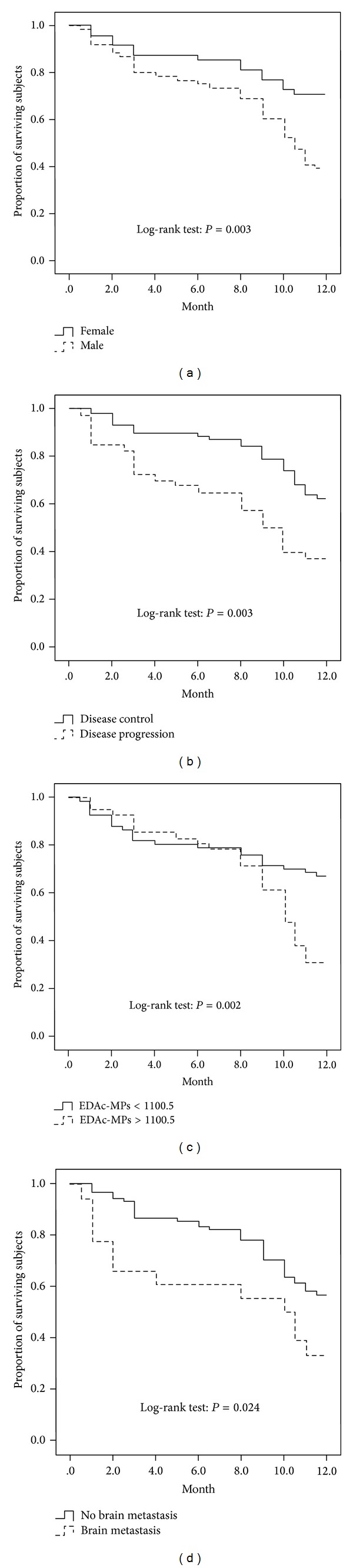
Kaplan Meyer survival curve (KMSC). (a) The KMSC showing significantly higher one-year mortality rate in male than in female in the same setting of end-stage of non-small cell lung cancer (ES-NSCLC). (b) The KMSC showing significantly higher one-year mortality rate in disease control than in disease progression. (c) The KMSC showing significantly higher one-year mortality rate in circulating level of (EDAc-) MPs ≥ 1100.5 counts/mL than that of this biomarker <1100.5 counts/mL. However, the statistical significance only occurred at late stage of the first year. (d) The KMSC showing significantly higher one-year mortality rate in presence than in absence of brain metastasis.

Kaplan Meyer survival curve (Figure 3(a)) demonstrated that the one-year mortality rate was significantly higher in male than in female in the same setting of ES-NSCLC. Additionally, this analytic method demonstrated that the one-year mortality rate was significantly higher in disease progression patients than in disease control patients (Figure 3(b)). Of particular importance was the fact that the statistically significant difference was found in the very early time point (i.e., at <2.0 months) of one-year follow-up. Furthermore, based on analysis of circulating level of EDAc-MPs that was obtained from the cut-off level of ROC, higher circulating levels of this biomarker (i.e., ≥1100.5 counts/mL) were found to be significantly associated with higher one-year mortality, although the significant difference was observed only at a relatively late time of the first-year follow-up (Figure 3(c)). Brain metastasis is an independent factor for predicting one-year survival outcome in ES-NSCLC patients; Kaplan Meyer method also demonstrated the statistical significance between brain metastatic patients and non-brain metastatic patients (Figure 3(d)).

**Figure 4:**
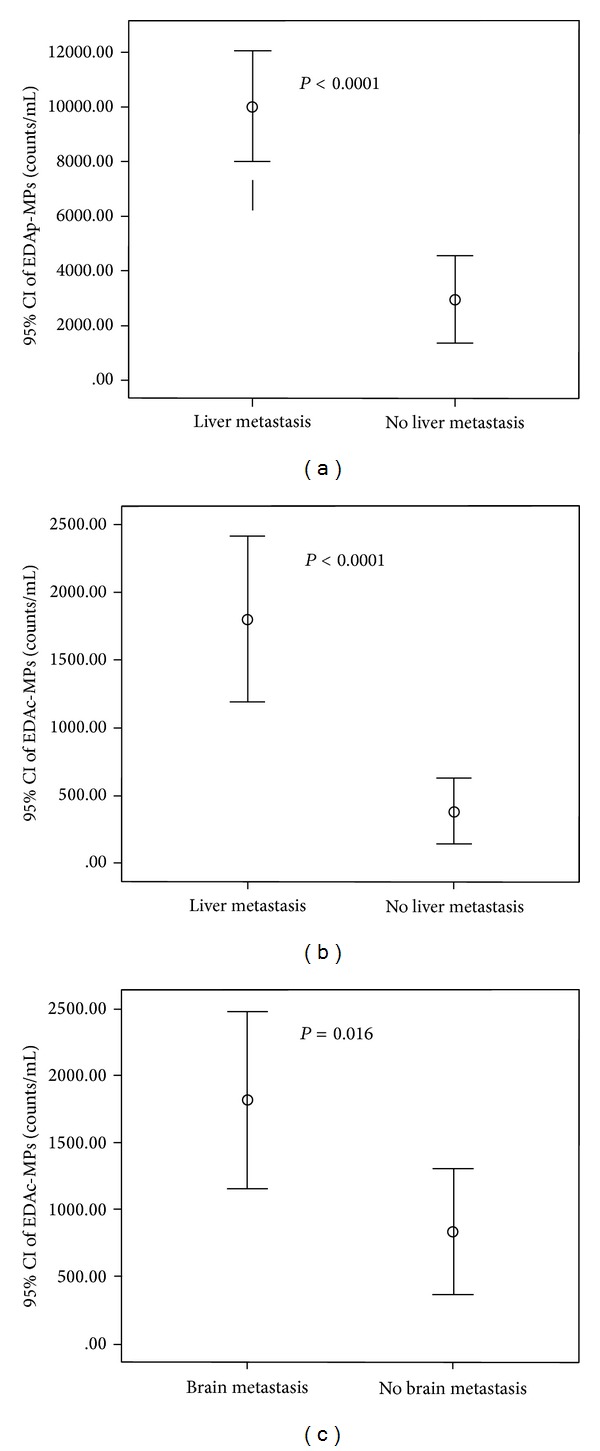
Positive relevance between microparticles and liver and brain metastasis. (a) Endothelial-derived apoptotic MPs (EDAp-MPs) were significantly predictive of liver metastasis. (b) Endothelial-derived activated MPs (EDAc-MPs) were significantly predictive of liver metastasis. (c) Endothelial-derived activated MPs (EDAc-MPs) were significantly predictive of brain metastasis. CI = confidence interval.

Liver and brain metastasis would lead to one-year mortality in ES-NSCLC patients; thus, we wished to see the relevance between liver and brain metastasis and four types of MPs. Figures 3(a) and 3(b) demonstrated that patients with liver metastasis would result in higher circulating EDAp-MPs and EDAc-MPs level than patients with non-liver metastasis.  Figure 4(c) also showed that patients with brain metastasis had higher circulating EDAc-MPs level than patients with non-brain metastasis. Circulating EDAc-MPs level not only is an independent factor predicting one-year mortality but also highly correlates with liver and brain metastasis in ES-NSCLC patients.

## 4. Discussion

This study investigated whether level of circulating MPs could be potentially useful as a biomarker in daily clinical practice to predict one-year mortality in ES-NSCLC patients. The findings yielded several clinical implications. First, surprisingly, the circulating levels of PDAc-MPs, PDAp-MPs, and EDAp-MPs did not differ between one-year survivors and one-year nonsurvivors. This finding, although contrary to our expectation, suggested that these three MPs might not be useful as biomarkers for predicting clinical outcome in the ES-NSCLC setting. Second, higher levels of circulating EDAc-MPs were significantly correlated to liver metastasis, disease progression after complete course of first-line treatment, and one-year mortality. Third, male gender was found to be a significant negative determinant of one-year survival. Fourth, increased circulating level of EDAc-MPs along with male gender and brain metastasis were significantly and independently predictive of one-year mortality.

Many studies have shown that circulating MP levels are remarkably increased in various diseases [14, 24–29]. In particular, levels of circulating MPs have been found to be markedly increased in hematological malignancies [28, 29], breast cancer [30], ovarian cancer [39], and colorectal cancer. However, in contrast with these findings [28, 29, 39], limited data is available regarding the association between circulating level of MPs and LC [31, 32], particularly in the ES-NSCLC setting [40]. Our recent study showed that the levels of four types of MP (i.e., PDAc-MPs, PDAp-MPs, EDAp-MPs, and EDAc-MPs) are significantly higher in LC patients than in healthy control subjects [32]. The present study which was designed to extend our earlier study [32] validated those findings, showing that, as compared to healthy control subjects in the study [32], the levels of the four circulating MPs are notably increased in ES-NSCLC patients.

Although radiotherapy and multiagent chemotherapy are used in the treatment of patients with ES-NSCLC, the prognosis remains poor, with a median survival of 6 to 12 months and 1-year survival rates between 20% and 50% [41, 42]. In the current study, the one-year survival rate was 52.3% (i.e., >50%). This finding shows that one-year survival rate in the present study was in line with the literature [41, 42].

A principal finding in the present study was that male gender is independently predictive of one-year mortality. Interestingly, the results from other recent large-sample size clinical trials [43, 44] do not support this finding. We remain uncertain about why our results are inconsistent with other recent studies [43, 44]. Perhaps the relatively small sample size in our study and ethnic difference between our patients and those of two recent studies [43, 44] could explain this discrepancy. Additionally, the EGFR tyrosine kinase has been reported to be expressed on the cell surface of a substantial percentage of NSCLCs [45]. Furthermore, the activating EGFR mutations are more commonly observed in patients with non-squamous cell carcinoma and no prior history of smoking, as well as in females and those of Asian descent [46]. These may be also the reasons for explaining our finding.

Microparticles are considered to be important biological effectors of several different physiological and pathological processes [47, 48]. There is increasing evidence not only of their role in haemostasis and thrombosis, but also of their importance in cancer cell survival, invasiveness, and metastasis [31, 40, 49]. One important finding in the present study was that incidence of liver metastasis was notably increased in one-year nonsurvivors in comparison with one-year survivors. In addition, the disease progression was also significantly higher in the former group of patients than in the latter group of patients. Importantly, brain metastasis which was significantly higher in one-year nonsurvivors than in one-year survivors was found to be independently predictive of one-year mortality. Our findings, therefore, reinforced the findings of the previous studies [31, 40, 49].

The most important finding in the present study was that an increase in circulating level of EDAc-MPs was an independent predictor of one-year mortality. Some recent studies have shown that high levels of MPs consistently correlate with cancer aggressiveness and poor prognosis [47–50]. Accordingly, our present study, in agreement with the prior studies [47–50], further suggests that circulating EDAc-MPs may represent a novel biomarker of LC disease activity and be a useful biomarker predictive of prognostic outcome in patients with ES-NSCLC. Surprisingly, one recent study [40] revealed that elevated levels of MPs were associated with longer survival in patients with ES-NSCLC. We remain uncertain about why these results contrast with ours [40]. We tentatively propose several reasons for the discrepancy. First, the sample size was larger in our study as compared with the previous study [40] (i.e., 107 patients vs. 60 patients) which could, at least in part, explain this different finding. Second, the methodology for measuring the circulating level of MPs was quite different in our study and the earlier study [40]. Third, the measurement of different MPs in the present study and the earlier study [40] could once again explain the different findings.

## 5. Study Limitations

This study was limited by the following factors. First, although the results are promising, the sample size of this study was relatively small; thus, the conclusions based on the findings of the present study are tentative and care should be taken in extrapolating the results of this study to the clinical setting. Second, the statuses of patients who were enrolled into the study were not completely identical at the enrollment time; that is, some patients had received treatment prior to presentation. Third, this study did not measure whether the “apoptotic” endothelial microvesicles contained the fragments of nuclei. Thus, the result of the study did not completely rule out that the fragments of nuclei also coexisted with membrane fragment of MPs during flow cytometric analysis. Finally, this study did not measure circulating level of the exosomes (derived from endosomal compartments that have size 30–100 nm). Therefore, we did provide the information for whether the exosomes were also an important predictor of prognostic outcome in patients with advanced stage of lung cancer.

In conclusion, we found that increased circulating level of EDAc-MPs was independently predictive of one-year mortality in setting of ES-NSCLC. This finding suggests that EDAc-MPs might have potential as a new biomarker for determining prognostic outcome in patients with ES-NSCLC after first-line treatment.

## Figures and Tables

**Table 1:** Baseline characteristics and clinically relevant variables of 107 study patients.

Variable	One-year survivors (*n* = 56)	One-year nonsurvivors (*n* = 51)	*P* value
Age (yrs)	64.9 ± 13.1	62.9 ± 11.2	0.393
Male gender % (*n*)	42.9% (24)	72.5% (37)	0.002
Body mass index (kg/m^2^)	22.8 ± 3.5	22.8 ± 3.0	0.986
History of smoking % (*n*)	41.1% (23)	54.9% (28)	0.127
Hypertension % (*n*)	54.9% (7)	23.5% (12)	0.135
Diabetes mellitus % (*n*)	7.1% (4)	13.7% (7)	0.341
Red blood cell count (×10^6^/dL)	4.1 ± 0.6	4.0 ± 0.7	0.514
White blood cell count (×10^3^/dL)	7.30 ± 2.55	8.17 ± 3.48	0.135
Platelet count (×10^4^/dL)	22.99 ± 8.91	25.59 ± 11.60	0.189
Creatinine (mg/dL)	1.06 ± 0.69	0.99 ± 0.43	0.487
AST (IU/L)	48.93 ± 26.66	43.55 ± 23.26	0.106
ALT (IU/L)	47.00 ± 22.74	40.65 ± 17.06	0.267
First-line adjunctive therapy % (*n*)			
Concurrent chemoradiotherapy	8.9% (5)	15.7% (8)	0.378
Traditional chemotherapy	50% (28)	66.7% (34)	0.116
Target therapy	41.1% (23)	17.6% (9)	0.011
Distant metastasis % (*n*)	64.3% (36)	70.6% (36)	0.542
Stage % (*n*)			1.000
IIIb	17.9% (10)	17.6% (9)	
IV	82.1% (46)	82.3% (42)	
Cell type % (*n*)			0.134
squamous cell carcinoma	21.4% (12)	35.3% (18)	
nonsquamous cell carcinoma	78.6% (44)	64.7% (33)	
Metastatic site % (*n*)			
Pleura	16.1% (9)	23.5% (12)	0.336
Lung	19.6% (11)	21.6% (11)	0.813
Bone	32.1% (18)	21.4% (16)	1.000
Liver	3.6% (2)	17.6% (9)	0.023
Adrenal gland	0% (0)	2.0% (1)	0.468
Brain	10.7% (6)	23.5% (12)	0.075
First-line treatment status % (*n*)			0.017
disease control	75.0% (42)	51.0% (26)	
disease progression	25.0% (14)	49.0% (25)	
Performance Status % (*n*)			0.042
0	23.2% (13)	25.5% (13)	
1	75.0% (42)	60.8% (31)	
2	1.8% (1)	13.7% (7)	
Charlson comorbidity index	6.8 ± 2.3	7.7 ± 1.9	0.027

Data expressed as mean ± SD or % (*n*) of patients.

AST: aspartate aminotransferase; ALT: alanine aminotransferase.

**Table 2:** Flow cytometric quantification of circulating MPs levels among the 107 study patients.

Variables	One-year survivors (*n* = 56)	One-year nonsurvivors (*n* = 51)	*P* value
CD31^+^CD42b^−^AN-V^+^ (counts/mL)∗	9604.47 ± 10879.55	8979.27 ± 8496.78	0.743
CD31^+^CD42b^−^AN-V^−^ (counts/mL)∗	942.33 ± 1556.86	2473.18 ± 3827.60	0.006
CD31^+^CD42b^+^AN-V^+^ (counts/mL)∗	30435.52 ± 29380.62	39718.43 ± 32105.38	0.118
CD31^+^CD42b^+^AN-V^−^ (counts/mL)∗	92391.74 ± 206545.58	48540.96 ± 100731.50	0.171
CEA^†^	45.59 ± 119.28	130.11 ± 456.11	0.177

*CD31^+^CD42b^−^AN-V^+^ = endothelial-derived apoptotic microparticles; CD31^+^CD42b^−^AN-V^−^ = endothelial-derived activated microparticles; CD31^+^CD42b^+^AN-V^+^ = platelet-derived apoptotic microparticles; CD31^+^CD42b^+^AN-V^−^ = platelet-derived activated microparticles.

^†^CEA: carcinoembryonic antigen.

**Table 3:** Predictors of 1-year mortality in non-small cell lung cancer patients by univariate analysis and multivariate logistic regression analysis.

Variable	Comparison	Univariate OR^b^ (95% CI^c^)	*P* value	Multivariate OR^b^ (95% CI^c^)	*P* Value
Gender	Male versus female	3.744 (1.671~8.391)	0.001	4.676 (1.542~14.175)	0.006
Liver metastasis	Yes versus no	6.000 (1.231~29.233)	0.027		
Brain metastasis	Yes versus no	2.667 (0.920~7.730)	0.071	5.378 (1.012~28.582)	0.048
First-line treatment status	Disease control versus progression	0.363 (0.162~0.811)	0.013		
Performance status	2 versus 0 & 1	9.935 (1.163~84.878)	0.036		
Charlson comorbidity index	Per 1 unit decrease	0.813 (0.674~0.981)	0.031		
CD31^+^CD42b^−^AN-V^−^ (counts/mL)^a^	Per 1 unit decrease	0.9996 (0.999~1.000)	0.021	0.9995 (0.999~1.000)	0.007

^a^CD31^+^CD42b^−^AN-V^−^ = endothelial-derived activated microparticle.

^b^Odds ratio.

^c^Confidence interval.
